# The Otoprotective Effect of Ear Cryotherapy: Systematic Review and Future Perspectives

**DOI:** 10.3390/audiolres12040038

**Published:** 2022-07-05

**Authors:** Dominik Péus, Shaumiya Sellathurai, Nicolas Newcomb, Kurt Tschopp, Andreas Radeloff

**Affiliations:** 1 Department of Otorhinolaryngology, University of Oldenburg, 26122 Oldenburg, Germany; nranewcomb@gmail.com (N.N.); andreas.radeloff@uol.de (A.R.); 2 Department of Otorhinolaryngology, Cantonal Hospital Baselland, 4410 Liestal, Switzerland; s.sellathurai@stud.unibas.ch (S.S.); kurt.tschopp@ksbl.ch (K.T.); 3 Department of Biomedicine, University of Basel, 4001 Basel, Switzerland; 4 The Software Revolution, Inc., Kirkland, WA 98034, USA

**Keywords:** otoprotection, cryotherapy, hypothermia, inner ear

## Abstract

This systematic review investigates ear cooling and cryotherapy in the prevention and treatment of inner ear damage and disease, within the context of animal models and clinical studies. A literature search was carried out in the databases Pubmed and Cochrane Library. Ten studies were identified concerning the otoprotective properties of cryotherapy. Nine of these were rodent in vivo studies (mice, rats, gerbils, guinea pigs). One study involved human subjects and investigated cryotherapy in idiopathic sensorineural hearing loss. The studies were heterogeneous in their goals, methods, and the models used. Disorder models included ischemia and noise damage, ototoxicity (cisplatin and aminoglycoside), and CI-electrode insertion. All ten studies demonstrated significant cryotherapeutic otoprotection for their respective endpoints. No study revealed or expressly investigated otodestructive effects. While limited in number, all of the studies within the scope of the review demonstrated some degree of cryotherapeutic, otoprotective effect. These promising results support the conducting of further work to explore and refine the clinical applicability and impact of cryotherpeutics in otolaryngology.

## 1. Introduction

Pathologic conditions of the inner ear manifest clinically in varying degrees of hearing loss, tinnitus, and dizziness. In some cases, these symptoms severely impair quality of life (QoL) and carry emotional and financial consequences for those affected and their relatives [1,2]. The pathophysiology of many inner ear diseases is not well-known; disorders with a known cause are somewhat better understood. The latter include blast injury, acoustic trauma, or ototoxic hearing loss [3,4]. In contrast, the etiology of disorders such as idiopathic sensorineural hearing loss and acute vestibular neuropathy remain unclear (Schick et al. 2001), although potential suspected causes include auto-immunological processes [5], viral infections [6,7], and vasomotor dysregulation [8]. An improved pathogenetic understanding of these disorders has only sometimes improved therapeutic outcomes. 

The two most common therapies for the disorders listed above are steroids and antioxidants; both deliver mixed results in the clinical setting. For example, steroids, despite being a well-established therapy for cisplatin ototoxic hearing loss, do not positively influence outcomes [9]; adverse effects include high blood pressure, blood sugar disorders, and decompensation of psychological comorbidities [10]. Despite substantial testing, the results with antioxidants such as sodium thiosulfate also remain mixed. The 2017 Freyer et al. randomized control trial of oncological diseases in children and young people treated with cisplatin employed an antioxidant containing sodium thiosulfate in the test group, as well as cisplatin [11]. This was regarded as the first effective therapy for cisplatin-induced hearing loss. However, a retrospective analysis demonstrated significantly worse QoL in the test group with advanced tumor disease [12]. As a result, a potential reduction by the antioxidant of cisplatin’s anti-cancer activity cannot be ruled out. At present, known otoprotective therapies are both limited in their availability and applicability, and demonstrate questionable efficacy.

In the search for better otoprotective therapies, clinicians and researchers are evaluating novel therapeutic modes. To this end, the otoprotective effect of cryotherapy (otoprotective hypothermia, OH) has been investigated since the 1980s, and a number of in vivo studies have been published. It is somewhat surprising that local cooling in otorhinolaryngology (with a few exceptions) has not been studied more actively, considering thermal manipulation of the inner ear is an established clinical routine in the diagnosis of the vestibular organ, and the broad protective effects of cryotherapy are included in intensive care medicine and oncology (prevention e.g., scalp hair loss during chemotherapy) daily routine [13,14,15].

In this review we seek to elucidate the following key topics: (i) the current state of application and understanding of cooling of the inner ear; (ii) the protective effect of cooling on hearing; (iii) the pros and cons of cryotherapy in human otolaryngology disorders; and (iv) potential methods of administering cryotherapy.

## 2. Methods

**Figure 1:**
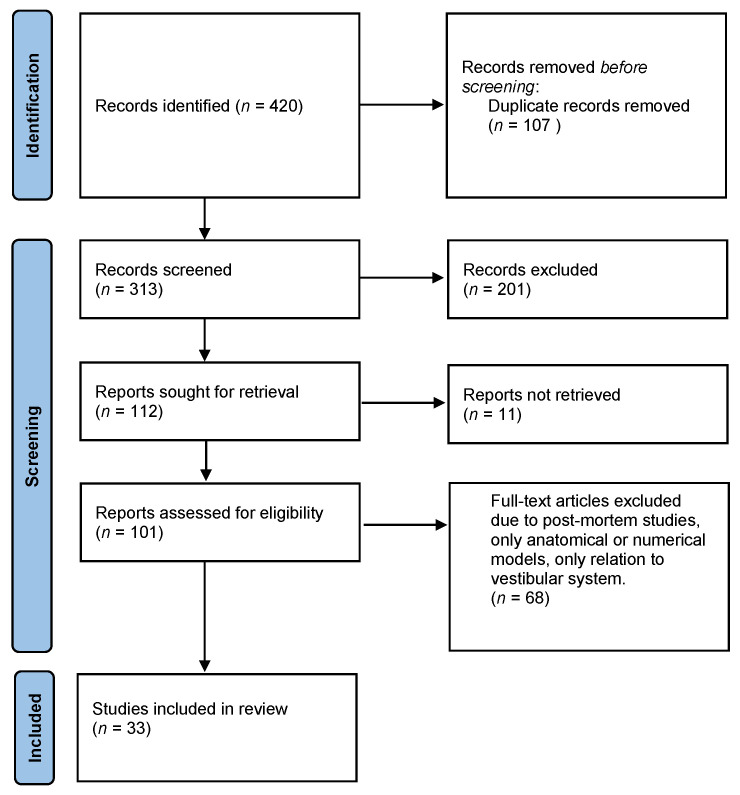
Flow chart of the study selection procedure.

A literature search was conducted between 1 September and 30 September, and again between 1 December 2021 and 31 January 2022, according to PRISMA guidelines [16]. Search parameters included (a) condition (e.g., hearing loss, deafness, blast injury, tinnitus, hair cell loss) and (b) intervention (e.g., hypothermia, cryotherapy, cooling). The terms and Boolean combinations were adapted for the database searches in Pubmed and the Cochrane Library. Figure 1 demonstrates the search outcomes as a PRISMA flow diagram. Literature cited within the included studies was also reviewed. No restrictions were placed on the date of publication. Studies from the search sample set described above were included in the review if they explored effects of cooling on the inner ear. 

For this review, two review authors (DP and SS) independently searched and identified eligible studies and trials based on the following characteristics: study population (clinical studies, or in vivo studies) and study intervention (clinical study recruiting probands with an inner ear disease receiving or not receiving cryotherapy; inner ear model of a cochlear damage with or without cryotherapy).

The review authors screened titles and abstracts to identify potentially relevant citations. The full text of the article was retrieved and reviewed when the title and abstract screening were ambiguous in their relevance. The review authors independently assessed the eligibility of the studies, by filling out eligibility forms designed in accordance with the review study inclusion criteria.

## 3. Results

The studies included in the review were heterogeneous in their objectives, the animal models used, how cold was applied, and measurement methods. A total of 33 studies were identified investigating the impact of cooling on the inner ear. However, only 10 studies of the 33 investigated the otoprotective potential of cryotherapy in the ear. The remaining 23 studies were more heterogenous and focused on fundamental research. 

### 3.1. Cooling Effects on Compound Action Potential

Fifteen of the twenty-three studies investigating the impact of cooling on the cochlea, without reference to otoprotection, used animal models. Five of these in vivo studies investigated the impact of cooling on compound action potentials (CAP) [17,18,19,20,21]. Brown et al. measured CAP in guinea pigs with whole-body hypothermia and used 1-ms rise–fall time to evoke the CAP. Small changes were observed when cooling to between 37 °C and 34 °C. With further cooling, amplitude, waveform, threshold, and latency changed greatly and mostly reversibly. The amplitude decrease in the high frequencies was explained by a selective decrease in the sensitivity of units tuned to higher frequencies. However, the increase in the latency of units was independent of their characteristic frequency. The authors presume that this could be explained by the fact that the conduction velocity of myelinated nerves decreases linearly with the decrease in temperature.

### 3.2. Cooling Effects on Endocochlear Potential and Single nerve Fibers

Cooling has been shown to reduce the endocochlear potential (EP) in the base of the guinea pig cochlea, from 86 mV to 73.2 mV on average, after 2 h of cooling, reaching a rectal temperature 29 °C [22]. Two studies investigated the impact of cooling on the endocochlear potential (EP), as facilitated by the stria vascularis [20,22]. Single auditory nerve fiber recordings were used by two studies, which suggested that mechanical properties were more changed in the base of the cochlea then in the apex. This further supports the validity of CAP and EP cooling findings [23,24].

### 3.3. Cooling Effects on Otoacoustic Emissions

Temperature dependence of distortion product (DPOAE) and transient evoked otoacoustic emissions (TEOAE) were found in at least three studies [25,26,27]. No changes were observable between 37 °C and 33–34 °C, but DPOAE and TEOAE were significantly, but reversibly, suppressed at temperatures below the lower range.

### 3.4. Other In Vivo Studies

Few studies used non-electrophysiological measures. Miller et al. published several papers using laser Doppler flowmetry to measure the cochlear blood flow in guinea pigs. They exposed the bone of the basal turn of the cochlea to a cryoprobe. This induced a reduction of the cochlear blood flow for cryoprobe temperatures of −40 °C compared to 0 and −10 °C [28,29].

The studies listed above prove that in vivo cooling leads to reversible changes of the different components of the inner ear, with a tendency to most strongly affect the base of the cochlea. However, investigations upon the cooling application itself were not systematic or closely comparable across studies.

### 3.5. Comparison of Cooling Administration

Only two in vivo studies were identified comparing different application methods of cooling. Smith et al. 2007 used a rat model and placed a temperature micro-probe in the basal flexion of the cochlea. Water irrigation over the external ear canal (EEC) was compared with an opened bulla approach [30]. EEC irrigation with water cooled to 14 °C and 11 °C decreased cochlear temperature on average by 1.1 °C and 1.6 °C, respectively. Bulla irrigation with water cooled to 14 °C and 11° decreased cochlear temperature on average by 3.3 °C and 4.1 °C, respectively. Stanford et al. 2020 compared EEC irrigation and a thermoelectrically cooled metal ear bar in a guinea pig model. The ear bar needed to be cooled to temperatures 7 °C lower than the EEC irrigation temperature, to achieve similar cooling effects [31]. These two studies demonstrated that cooling applied locally over the external ear canal leads to temperature changes within the inner ear in vivo. In contrast, most other in vivo studies used whole-body hypothermia. Localized cooling methods are more interesting, clinically, than whole-body hypothermia, due to the more practical route to clinical application.

### 3.6. Cooling-Induced Otoacoustic and Temperature Changes in Human Studies

Temperature measurements in the tissues, cells, or fluid compartments of the inner ear in living human subjects are lacking. As a result, surrogate measurements such as otoacoustic emissions were used in five studies. During cardiac surgeries whole-body hypothermia was applied. It was shown that the TEOAE and DPOAE response is dependent on the body core temperature [32,33,34,35,36].

Two human studies measured induced temperature change on the surface of the bony labyrinth [37,38]. Kleinfeld and Dahl measured temperature variations at the lateral bony canal during 11 ear surgeries (posterior tympanotomies). Cooling was applied by filling up the external ear canal with about 2 ml 20 °C tempered or 0 °C tempered water. The mean temperature decrease was 0.8 °C and 1.2 °C within 70 s. However, the administration of only 2 mL cooling fluid is not likely to be sufficient to cool down the inner ear. What remains unclear is what temperature change can practically be provoked in living human subjects with EEC cooling or neck cooling, nor are the methods described that explain how temperature changes should be measured within the inner ear in living human subjects without the need for surgery.

### 3.7. Evidence of Cryotherapy for Prevention and Therapy

Ten studies investigating the otoprotective effects of cooling were identified. Of these, nine were in vivo studies with rodents; one was a clinical study of hearing loss in human patients. Table 1 summarizes the included papers, sorted by investigated effect and localization of cooling.

*Decrease in cisplatin ototoxicity*: Two studies by the Spankovich group demonstrated protection of hair cell activity in guinea pigs under the application of non-invasive cooling of the outer ear canal [31,39]. Spankovich et al. irrigated the outer ear canal for 20 min with 30 °C water, 2 h before administering a single dose of cisplatin (12 mg/kg, i.p.). The same research group refined their methods for a later 2020 study and used a more accurate cisplatin dosing regimen of one application per week (4 mg/kg, i.p.) for 3 weeks, which cumulatively reached dose equivalence with Spankovich et al. (12 mg/kg) [31]. The results of Spankovich and Stanford did not significantly differ. Cisplatin exposure over time resulted in minimal, insignificant ABR and DPOAE measurement threshold shifts for the control and intervention groups. After the second cisplatin dose, there was a significant threshold shift in the control group, a small, nonsignificant threshold shift in the intervention group, and no significant difference between the two groups. After the third dose, a significantly greater threshold shift was shown in the control group compared to the cooled group. In addition, hair cell loss in the cochlear basal region was significantly reduced for the intervention group [31]. In short, pre-emptive cryotherapy was successfully demonstrated to protect hearing and hair cells from repeated cisplatin exposure.

*Cranial cooling in the treatment of idiopathic hearing loss*: While the etiology of idiopathic sensorineural hearing loss has not been clarified, many forms of therapy have been tested. Hato et al. conducted a multi-center study [40], motivated by the otoprotective effect of hypothermia observed in the ischemia model of Watanbe et al. [41]. The patients in Hato et al.’s study hypothermic group (N = 86) were admitted and treated with a cooled water pillow for 48 h, in addition to standard treatment for 7 days, which was 60 mg prednisolone for days 1–3 and reduced doses days 4–7, and 60 mg of Adenosine Triphosphate (ATP) and 150 mg of methylcobalamin over 7 days. The water pillow was cooled to 15 °C and was changed 4–5 times per day. The patients used the water pillow for the first 48 h after admission, with restricted activity. The control patients received only the medications. No adverse effects of the cooling pillow therapy were observed. At 6 months, there was no observable statistical case-matched analysis difference in patients aged 60 years or older. The below 60 years of age subgroup showed a statistically significantly higher recovery rate, i.e., 71.4% vs. 46.5% in the control group [40]. Complete recovery was defined as hearing level recovered within 20 dB at five frequencies (0.5, 1, 2, 4, and 8 kHz) or hearing level recovered to that of the contralateral, unaffected ear.

*Increasing cochlear ischemia tolerance as a model for idiopathic hearing loss*: Some animal studies model idiopathic hearing loss by disconnecting blood supply to the cochlea. These studies showed that mild full-body hypothermia (32 °C) reduced and usually also prevented hearing loss, as measured in terms of brain stem audiometry and immunohistochemistry (number of intact hair cells) when compared to the normothermic group [41,42,43,44].

*Reduction of the destructive effect of acoustic trauma*: Henry et al. demonstrated the protective effects of cryotherapy within the context of acoustic trauma in mice and gerbils [45]. They exposed mice and gerbils of different ages to noise (5 min, 8–16 kHz octave band, 115 dB SPL). They then measured the cochlear action potential, before and after noise exposure, at 4 days. The damage was quantified as a threshold shift in dB. The average permanent threshold shift (PTS) was 37.2 dB in young mice in the normothermic group. In the mild hypothermic group (30 °C) the average PTS was 26 dB. An electron micrograph of the apical, mid, and basal parts of the cochlea was taken. The authors asserted that the physiological findings support CAP measurements, without providing a further quantitative analysis.

*Increasing residual hearing after cochlear implantation (CI)*: Balkany et al. conducted a rat-based animal study demonstrating improved residual hearing after CI in animals treated with systemic hypothermia (34 °C) [46]. They investigated the effect of mild hypothermia on hearing loss due to electrode insertion trauma in normal hearing rats. Electrode insertion leads to cochlear damage, which is clinically relevant in functional deaf patients with residual hearing, i.e., at low frequencies. ABR threshold shift was substantially reduced in the hypothermia group (−4.2 dB SPL) vs. the normothermia group (−15.9 dB SPL). Similarly, Tamames et al. demonstrated significant otoprotection in a hypothermia group, quantified as reduced ABR shift and improved hair cell count [47]. The main difference between the Tamames and Balkany studies was in the application of cooling. Balkany et al. used mild whole-body hypothermia; Tamames et al. explored cold application to the cochlear promontory via a rod-like device.

## 4. Discussion

The research, conclusions, and ultimately cryotherapy’s clinical usefulness as a therapeutic approach remain a fragmented picture. In vivo study results are promising, and positive physiological effects in the inner ear were observed, but this evidence pertains mostly to full-body hypothermia, which is impracticable for use in clinical studies, and probably impractical in most clinical settings. The reviewed works applied thermal energy eclectically, so while the methods do not strongly corroborate one another, the results are still generally promising. Furthermore, the varied studies show multiple clinically interesting hypothermic impacts on the inner ear. Cryotherapy applications variously increased ischemic tolerance, reduced cisplatin ototoxicity, and reduced both acoustic trauma and residual hearing loss as caused by the CI electrodes. Nevertheless, little is known about the mechanisms that take place within the inner ear in hypothermic conditions. Taken together, these studies and measurements demonstrated that hypothermia has an effect, and frequently enough a beneficial effect; however, it remains unclear how that effect unfolds, and corroborating studies would be advisable to prioritize the form of application and verify preliminary results in the areas where doing so would have the greatest impact; i.e., QoL impacting clinical areas where other therapies are lacking or where otological damage is particularly severe.

Most of the in vivo studies had a relatively simple design and relied upon a combination of hypothermic exposure in combination with different potentially hearing-harming conditions and electrophysiological measurement, e.g., a hair-cell count. These captured measurable changes, but were not generally sufficiently precise in application, to ascertain what methods of application affect electrophysiological change most effectively, or why. Several studies investigated electrophysiological changes in CAP, CP, and OAE, and thereby demonstrated hypothermic effects at the basal part of the cochlea. CAP amplitude, waveform, threshold, and latency changed significantly, and mostly reversibly, below 32–34 °C (body core temperature) [17]. In the case of the cisplatin-model the main mechanism is, in all likelihood, reduced blood flow and, therefore, a reduced uptake of ototoxic substances, e.g., cisplatin [48]. Additional mechanisms of action might include slowed metabolism, reduced oxidative stress, and the involvement of cold shock proteins [49]. Higher metabolic and mechanical activity might explain the higher susceptibility of high-frequency areas vs. low-frequency areas of the cochlea.

It is worthwhile to further explore the theoretical physiology of cooling. Cooling reduces the blood flow of the stria vascularis and, therefore, impacts the endocochlear potential, but it is unclear whether the vasoconstriction (and therefore a reduced blood flow) on the level of the cochlea is responsible for some of the reported therapeutically promising observations, or if further systemic changes within the body (e.g., reduced cardiac output following whole-body hypothermia) are responsible for the effect [22,29].

Furthermore, not all the physiological phenomena are explained by processes in the organ of Corti or the stria vascularis, because increased CAP latency was also observed. This implies a hypothermia-associated process in the first neuron. This simply reinforces the lack of clarity as to whether slowed metabolism or reduced perfusion, or perhaps neither or both, are responsible for neural changes. Future research regarding physiological changes of the inner ear should use models that more precisely localize cooling at the ear, avoiding full body hypothermia, to differentiate better local cooling effects and exclude more general processes. Furthermore, methods should be applied that support the investigation of hypothermia-associated changes on an intracellular level with a differentiation between the different cell types, to further uncover the precise mechanism by which therapeutic effects are being achieved (or to hint at potentially undesirable side-effects).

There are a number of otologic diseases currently lacking reliable therapy options, and which are potential targets for cryotherapy. Within this context, at present, the state of the available literature implies that there is no consensus related to the prioritization of which pathological condition or conditions might be best suited for study. In terms of the most applicable therapeutic methods, only neck-applied cooling pad otoprotective hypothermia studies have been conducted in humans, and these were entirely limited to idiopathic hearing loss [40]. It is questionable whether idiopathic hearing loss is the most relevant application for cryotherapy in otology disorders, and whether neck cooling is a sufficient therapy for the disorder. Idiopathic sudden hearing loss is difficult to investigate because of the unclear, possibly diverse, etiologies and the resultant variable time frames at which patients should be enrolled in a study. A further obstacle is the high spontaneous remission rates. The single study exploring otoprotection in humans by Hato et al. is promising but is insufficient for actionable, clinical conclusions to be drawn, related to the protective or therapeutic potential of cryotherapy in otorhinolaryngology.

Cisplatin-induced hearing loss might be a reasonable candidate for translational research with cryotherapy, because the etiology is well understood [12]. Further arguments for prioritizing cryotherapy for cisplatin-induced hearing loss, include the fact that the ototoxic agent is known (which improves quantifiable evaluation, due to the dose-dependency of the ototoxicity). Studies could be schedulable to consistent time frames in the ototoxic progression (time of drug administration), and a study group is clearly available as control, as cisplatin is generally ototoxic in healthy-hearing patients.

Other disorders, which have not been explored in this work, and for which studies were not discovered in our review (and presumably do not exist), but which also are potential cryotherapy candidates, include vestibular neuritis, zoster oticus, and middle ear infection with inner ear involvement. A characterization of the prevalence of these ailments, the scope of otological damage, and a review of the therapies generally available for these conditions would be useful in identifying which of these might be most suitable for exploring the usefulness of cryotherapy in care. These diseases were, of course, outside the scope of this review, because, to date, cryotherapy has not been attempted (or at least not attempted and published). A reasonable cryotherapeutic trial protocol, may be an accessible and efficient path to new QoL improvements for these disorders.

Besides prioritizing the best disease targets for future study, the cryotherapeutic protocol (mentioned above) seems to be a prime target for optimization and standardization. In short, the method of cooling that is best for otoprotective therapy has not yet been established. Disease targeting is inextricably connected to the question of how cryotherapy is applied to the human body. Hato et al. used neck cooling to ectopically cool. However, the approach of neck cooling is not new and is under debate for brain protection, e.g., out-of-hospital reanimations. Neck with full-head cooling was used in healthy volunteers and intensive care unit patients [50,51,52], demonstrating a temperature drop at the tympanic membrane of around 0.8–1.5 °C, with a significant reflexive increase in blood pressure. Sole use of neck cooling is probably not ideal for future cryotherapy trials, because of its potential inefficiency and side-effects.

Ear canal cooling seems practicable as a therapeutic vehicle. Cooling takes place close to the target organ (the inner ear) and would not obviously cause severe or unpredictable effects. Two studies were identified in which induced temperature change was measured in humans, and where the EEC was inundated with water [37,38]. Spankovich et. al. used EEC cryotherapy to reduce cisplatin toxicity in guinea pigs [31,39]. Notably, no human otoprotective study with an EEC approach seems to have been conducted yet. However, the procedure is basically known, is used, and is safe. Simultaneous bilateral application of caloric stimulation is a known and proven method. Studies have investigated its *diagnostic* value for the testing of the vestibular system and also central vestibular lesions [53,54,55]. Bilateral *therapeutic* cryotherapy should, therefore, also be feasible.

With regards to long-term cooling methods, due to the lack of a suitable long-term cooling device, there is no data available to conclude whether longer cooling durations of, for example, an hour or longer, are acceptable. Long-term cooling, in any case, would not seem to be a practical implementation.

Besides duration, the degree of cooling that is appropriate has also not yet been established. Mild local hypothermia may prove tolerable but insufficient to achieve effective otoprotection. This too requires exploration, though general cryotherapeutic studies should be useful to establish safe parameters for such explorations.

Through a focus upon cryotherapy for ototherapeutic purposes, it should be possible to arrive at reasonably standardized methods, which can better help to elucidate the otoprotective mechanisms and applications relevant to further develop new, useful, and cost-effective therapeutic applications for ototoxic disease.

## 5. Conclusions

Otoprotective cryotherapy is a therapeutic approach that has been proven by various in vivo studies. Although the studies were quite heterogeneous in terms of methodology and objectives, they demonstrate protective effects and carry clinical potential. Future in vivo studies on otoprotective cryotherapy should investigate molecular–genetic, metabolic, and cellular mechanisms, in addition to verifying otoprotective key outcomes (i.e., ABR and hair cell count). A better understanding of these mechanisms in all cells of the inner ear, including hair cells and support cells, as well as the stria vascularis, would help to guide future development of therapeutic methods, regardless of whether these are cryotherapy-oriented or not. Data on the tolerability and safety of bilateral, simultaneous cold application would be desirable, as such “soft” factors determine the acceptance and, thus, success in patients. Significant research effort is required to empirically provide grounds for clinical targets, methods, and recommendations. We recommend beginning such research in the diseases best understood, and then leveraging such useful principles as can be proven to drive further scientific and clinical progress.

## Figures and Tables

**Table 1:** Study characteristics.

Results	Whole-Body	Ectopic, Cervical	Invasive, Promontory	Ear Canal
Increase of the cochlear ischaemic tolerance	Takeda et al. 2008 (gerbils)			
	Watanabe et al. 2001 (gerbils)			
	Hyodo et al. 2001 (gerbils)			
Decrease of noise damage	Henry et Chole 1984 (mice, gerbils)			
	Henry 2003 (mice)			
Increasing the residual hearing after CI	Balkany et al. 2005 (rats)		Tamames et al. 2016 (rats)	
Increased rate of cured patients after sudden idopathic hearing loss		Hato et al. 2010 (human)		
Decrease of cisplatin ototoxicity				Spankovich et al. 2016
				Stanford et al. 2020
				(guinea pigs)

## Data Availability

Not applicable.
